# Effect of Water–Solid Ratio on the Performance, Microstructure Evolution, and Low-Carbon Characteristics of Multi-Solid-Waste-Based Flowable Stabilized Soil

**DOI:** 10.3390/ma19112247

**Published:** 2026-05-26

**Authors:** Jiaojiao Ni, Qing Jiang, Qiwei Zhan, Haitao Hu, Yongqi Zhao

**Affiliations:** 1 China Construction Industrial & Energy Engineering Group Co., Ltd., Nanjing 210046, China; 2 School of Civil Engineering and Architecture, Jiangsu University of Science and Technology, Zhenjiang 212100, China; 3 School of Transportation, Southeast University, Nanjing 211189, China; 230259390@seu.edu.cn

**Keywords:** flowable stabilized soil, water-solid ratio, mechanical properties, workability, microscopic mechanism, environmental benefits

## Abstract

To promote the high-value utilization of industrial solid wastes and address the disposal of excavated soils, a novel low-carbon composite cementitious material, solid waste-based geopolymer cement (SGPC), was developed, consisting of soda residue (SR), granulated blast furnace slag (GGBS), phosphogypsum (PG), and ordinary Portland cement (PC) in a mass ratio of 10:81:9:25, with industrial solid wastes accounting for 80% of the binder. The effects of water-to-solid ratio (W/S = 0.41–0.49) on the workability, mechanical performance, and microstructural evolution of SGPC-stabilized soil were systematically investigated to provide a sustainable alternative to conventional cement-based stabilizers. The results indicate that the optimum water-to-solid ratio is 0.43 (SGPC43), with a 28-day unconfined compressive strength of 1450 kPa, exceeding the engineering requirement of 0.8 MPa and reaching over 85% of that of a pure cement system (C43). The flowability remained 163 mm after 60 min, with initial and final setting times of 43 h and 58 h, respectively. Microstructural analysis revealed that the alkalinity provided by soda residue promotes the hydration of slag and phosphogypsum, forming interwoven calcium (alumino) silicate hydrate (C–(A)–S–H) and ettringite (AFt), which fill pores and form a dense structure, thereby enhancing mechanical performance. Environmental and economic assessments show that the CO_2_ emission of SGPC43 per ton of binder decreases from 930 kg CO_2_-e/t to 235 kg CO_2_-e/t (approximately 74.7% reduction), while the material cost decreases from 110 USD/t to 53 USD/t (approximately 51.8% reduction). A simplified uncertainty analysis indicates that the carbon reduction remains at 70% ± 5% and the cost reduction at 50% ± 5%, confirming the robustness of the results. Overall, SGPC43 demonstrates excellent engineering performance, environmental benefits, and economic feasibility, highlighting its potential as a low-carbon and sustainable stabilizing material.

## 1. Introduction

Flowable stabilized soil (FSS) is prepared by mixing soil, stabilizer, and water. Before hardening, the mixture exhibits excellent pumpable flowability, while after solidification it can satisfy engineering requirements such as strength, impermeability, and long-term stability. Therefore, FSS has been widely applied in construction scenarios with limited space and difficult material transportation, such as deep and narrow trenches, underground utility corridors, and culverts [1,2,3]. With the rapid advancement of global urbanization and the continuous expansion of transportation, hydraulic, and municipal infrastructure construction, a large amount of engineering waste soil (WS) is generated. FSS can be compared with conventional backfill materials such as compacted soil, cemented soil, and mortar-based backfill, offering superior flowability and construction efficiency, but exhibiting relatively low strength and high sensitivity to mix design. In China, approximately 80% of WS is still disposed of through stockpiling, with limited effective resource utilization [4,5]. In contrast, many developed regions have gradually shifted from conventional disposal practices toward resource-oriented management. In several European countries, driven by circular economy policies and sustainable construction strategies, waste soil has been widely reused in land reclamation and infrastructure projects [6]. Similarly, Japan has established a relatively mature management system for construction excavated soils through strict classification and recycling frameworks, significantly reducing reliance on final disposal [7]. However, in many developing regions, stockpiling and landfilling remain the dominant disposal methods due to insufficient recycling systems and policy support [6]. Overall, these comparisons indicate that China still faces significant challenges in improving the resource utilization of waste soil.

Therefore, improving the engineering performance of WS through chemical stabilization and realizing its resource utilization is of great significance. As traditional inorganic cementitious material, Portland cement (PC) has been widely used for soil stabilization due to its excellent engineering performance and economic advantages. During the hydration process, PC forms dense gel products that bind soil particles and fill internal pores, thereby significantly enhancing the mechanical properties of stabilized soils [8,9,10,11,12]. However, cement production is associated with high energy consumption and considerable carbon emissions. It is estimated that approximately 0.66–0.82 t of CO_2_ is emitted for every ton of cement produced, which imposes substantial environmental pressure [13,14,15]. Meanwhile, with the rapid development of modern industry, large quantities of industrial solid wastes have been generated. Among them, ground granulated blast furnace slag (GGBS), phosphogypsum (PG), and soda residue (SR) are produced in massive amounts, yet their comprehensive utilization rates remain relatively low. Improper disposal of these wastes may lead not only to serious environmental pollution but also to significant resource waste [16,17,18]. Consequently, partially or completely replacing PC with industrial by-products in soil stabilization has become an important approach for achieving carbon peaking and carbon neutrality targets [19,20].

GGBS is an important by-product of the ironmaking industry and contains reactive Ca and Si components with potential hydraulic activity [21,22]. Under alkaline conditions, the glassy structure of slag undergoes depolymerization, releasing active Ca, Si, and Al species. These species subsequently react to form hydration products such as C–S–H and C–A–S–H gels, which can significantly improve the mechanical properties and microstructural compactness of the matrix [23,24]. However, the hydration rate of GGBS is relatively slow, and alkaline activators are usually required to enhance its reactivity [25,26,27]. In recent years, GGBS-based cementitious materials have attracted increasing attention in soil stabilization. The hydration products generated from slag can effectively bind soil particles and fill pores, thereby improving the strength and stability of stabilized soils [28,29]. For example, Du et al. reported that a stabilizer mainly composed of carbide slag (CCR) and GGBS significantly improved the strength and impermeability of lightweight soil, exhibiting better performance than ordinary cement-stabilized soil [30]. Li et al. reported that the CS–GGBS system can effectively suppress water-induced volumetric expansion and structural damage, enabling the specimens to maintain a low swelling level (<0.3%) and limited strength loss [31]. During soda ash production via the ammonia–soda process, a large amount of solid by-product known as SR is generated, with a production coefficient of approximately 0.3 t/t [32,33]. Currently, SR is mainly disposed of through stockpiling, which poses considerable environmental risks [34]. Nevertheless, recent studies have shown that SR possesses strong alkalinity and can act as an activator to enhance the reactivity of supplementary cementitious materials. For instance, SR can promote hydration reactions in GGBS-based systems and thereby improve their mechanical properties [35]. Qi et al. reported that when NaOH and SR were jointly used as activators for GGBS, a mine backfill material with a 28 d compressive strength of 2400 KPa could be produced [36]. PG is the major solid by-product generated during the wet-process production of phosphoric acid. Its primary component is calcium sulfate dihydrate (CaSO_4_·2H_2_O), together with small amounts of phosphorus and fluorine impurities. With the rapid development of the phosphorus chemical industry, the accumulation of PG has become a major environmental concern [37,38,39]. Previous studies have shown that phosphogypsum (PG) can act as a sulfate-based activator in cementitious systems by supplying SO_4_^2−^ ions, which react with aluminate-bearing phases to form ettringite (AFt), thereby contributing to early structural development [40,41,42]. This sulfate-driven mechanism differs from classical alkali-activated systems governed by hydroxide-induced dissolution and aluminosilicate gel formation. Gu et al. used a mixture of 10% PC, 3% PG, 20% fly ash, and 8% quicklime to stabilize loess, achieving a compressive strength of 186,400 KPa at 3 d [43]. Zhang et al. reported that incorporating PG into cement systems significantly improved the strength of stabilized soils and optimized their pore structure [44]. In addition, Lu et al. demonstrated that the application of PG could improve the quality of alkaline soils [45], while Zeng et al. found that PG incorporation in cement–PG systems significantly enhanced the unconfined compressive strength and stiffness of dredged soils [46].

Although industrial solid wastes have been widely utilized in soil stabilization applications, existing studies have mainly focused on specific activation systems or individual performance evaluations, while systematic understanding of the performance evolution and underlying mechanisms in multi-source industrial solid waste synergistic systems remains limited. Particularly in GGBS-based stabilization systems, the structural formation mechanisms involving the synergistic participation of multiple industrial by-products, as well as the influence of water-to-solid ratio on macroscopic engineering properties and microstructural evolution, have not been sufficiently investigated. To address these research gaps, this study developed a multi-source industrial solid waste-based fluidized solidified soil system (SGPC) composed of SR, GGBS, PG, and PC. By adjusting the water-to-solid ratio within the range of 0.41–0.49, the effects on workability, mechanical properties, and microstructural evolution of the fluidized solidified soil were systematically investigated. Compared with previous studies, this work expands the research scope of multi-source industrial solid waste synergistic fluidized solidified systems and further elucidates the influence of water-to-solid ratio on fresh properties, mechanical performance, and microstructural pore evolution. Furthermore, the structure–property relationship of the multi-source solid waste system was explored from the perspectives of engineering performance, microstructural mechanisms, and low-carbon characteristics. The findings of this study contribute to a deeper understanding of the interaction mechanisms within multi-source solid waste-based cementitious systems and provide a theoretical basis and technical reference for their future research and engineering applications in geotechnical engineering.

## 2. Materials and Methods

### 2.1. Materials

**Figure 1:**
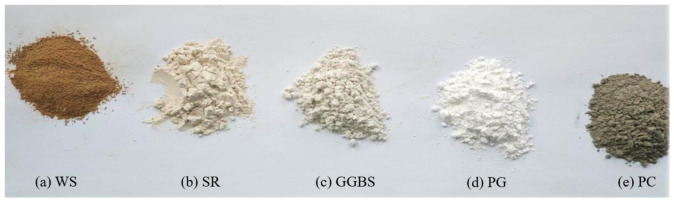
Physical morphology of raw materials.

**Figure 2:**
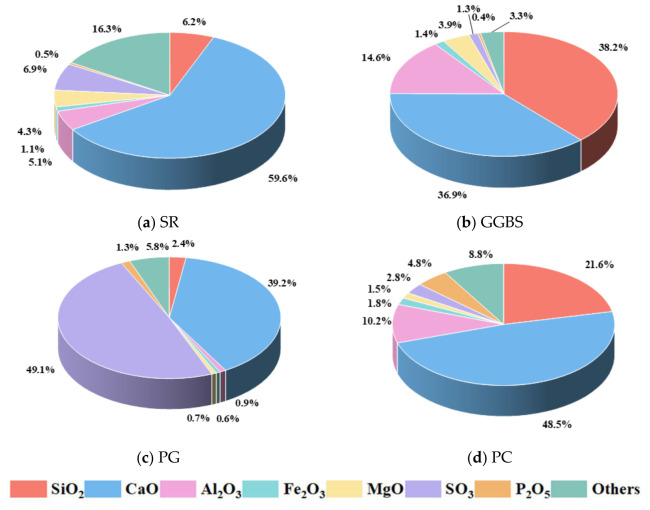
Chemical composition of raw materials/%.

**Figure 3:**
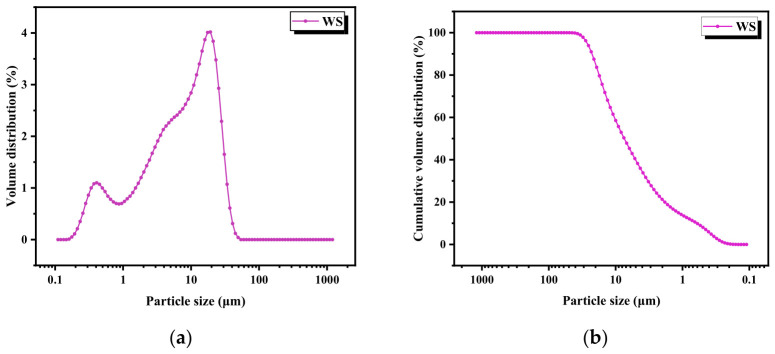

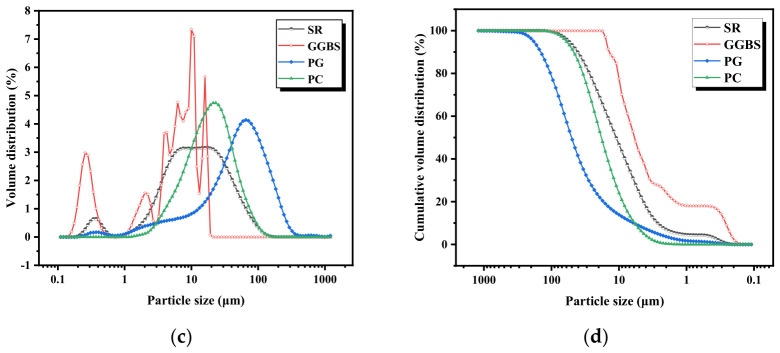
Particle size distribution curves of raw materials. (**a**) WS particle size distribution by volume; (**b**) WS cumulative volume distribution; (**c**) SGPC particle size distribution by volume; (**d**) SGPC cumulative volume distribution.

**Figure 4:**
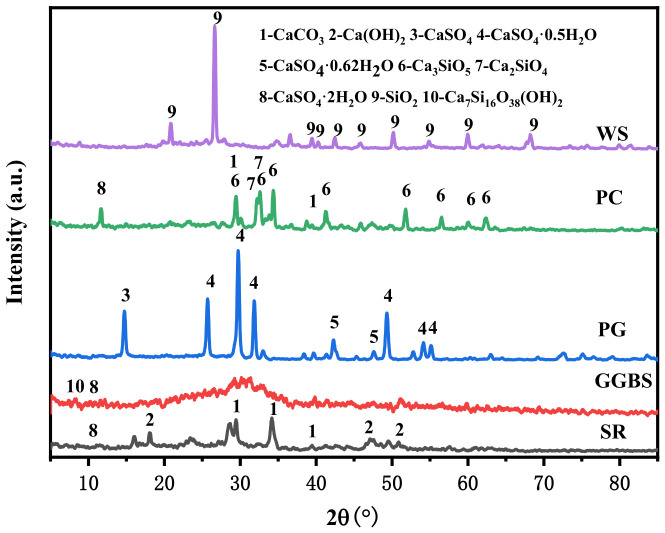
XRD analysis of raw materials.

**Figure 5:**
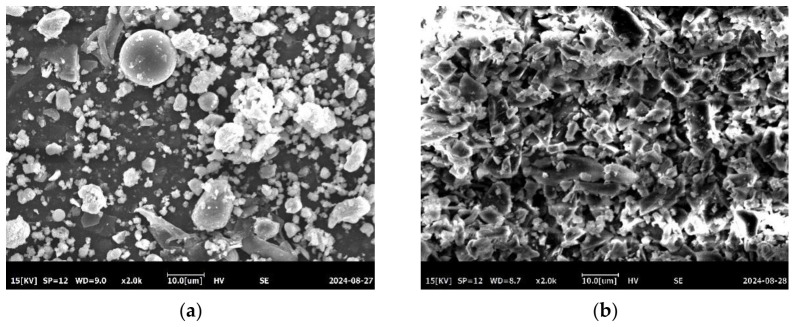

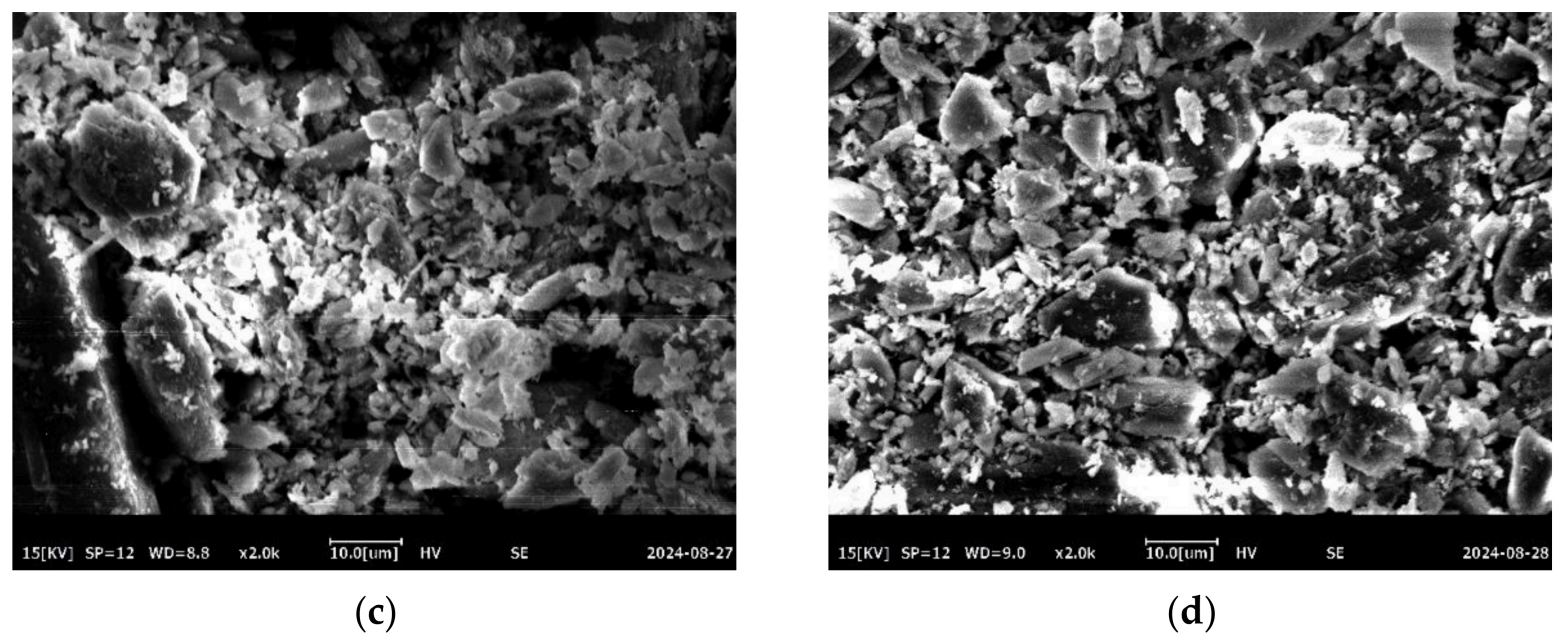
SEM images of the components of SGPC: (**a**) SR, (**b**) GGBS, (**c**) PG, (**d**) PC.

WS used in this study was collected from a construction site in Zhenjiang, Jiangsu Province, China, at a depth of 5–8 m. The soil appeared yellowish-brown and contained small amounts of fine gravel and plant root residues. Before testing, the WS samples were oven-dried at 105 °C to constant mass, crushed, and sieved through a 1 mm mesh to obtain relatively uniform particles. Prior to this treatment, the waste soil was first air-dried at room temperature to remove free moisture. To further improve sample uniformity, the sieved soil was subsequently ground using a ball mill for subsequent experiments and particle size distribution analysis. The basic physical properties of WS, including water content, liquid limit, plastic limit, plasticity index, pH value, and specific gravity, were determined according to the Standard for Geotechnical Testing Method of Highway Engineering (JTG 3430–2020) [47], and the results are summarized in Table 1. The stabilizing materials included SR, GGBS, PG, and PC. SR was obtained from a soda ash plant in Gongyi, Henan Province, China, with a specific surface area of approximately 435 m^2^/kg. GGBS (S105 grade) was supplied by a building materials company in Zhengzhou, Henan Province, China, with a specific surface area of about 582 m^2^/kg. PG was collected from a phosphochemical enterprise in Dongguan, Guangdong Province, China, with a specific surface area of approximately 345 m^2^/kg. PC (P.O 42.5) was produced by Hunan Conch Cement Co., Ltd., Changsha, China, with a relative density of 3.15 and a specific surface area of about 360 m^2^/kg. The morphological characteristics of WS and the stabilizing materials are shown in Figure 1. Their chemical compositions were determined by X-ray fluorescence (XRF) analysis (Figure 2). The particle size distribution curves measured by laser particle size analysis are presented in Figure 3. The mineralogical compositions were analyzed using X-ray diffraction (XRD), as shown in Figure 4. Quartz (SiO_2_, PDF#99-0088) was identified as the dominant mineral phase in WS. PG mainly consists of hemihydrate gypsum (CaSO_4_·0.5H_2_O, PDF#41-0024). Typical clinker minerals, including tricalcium silicate (C_3_S, PDF#49-0442) and dicalcium silicate (C_2_S, PDF#29-0329), were detected in PC. SR mainly contains calcium hydroxide (Ca(OH)_2_, PDF#44-1481) and calcium carbonate (CaCO_3_, PDF#05-0586), while GGBS exhibits a broad hump in the diffraction pattern, indicating its predominantly amorphous structure. The microstructures of the stabilizing materials were further characterized by scanning electron microscopy (SEM), and the corresponding images are shown in Figure 5. GGBS particles exhibit relatively smooth surfaces with irregular shapes, whereas PG particles mainly present plate-like morphologies. SR particles display a loose and porous structure, while PC particles generally show rough and angular surfaces.

### 2.2. Experimental Design and Specimen Preparation

Pre-treated WS described in Section 2.1 was used as the base material for preparing stabilized soil specimens. In this study, the composite stabilizer SGPC was incorporated at 14% of the dry mass of WS, which was determined based on preliminary optimization tests. To investigate the influence of water content on the performance of stabilized soil, five water-to-solid ratios (W/S) were considered: 0.41, 0.43, 0.45, 0.47, and 0.49. In addition, a control mixture stabilized with ordinary Portland cement was prepared at a W/S ratio of 0.43 to provide a reference for performance comparison. The selected W/S range (0.41–0.49) was determined based on previous studies on flowable cementitious materials and preliminary trial mixing to ensure adequate workability and avoid segregation or insufficient fluidity. Among them, the water-to-solid ratio (W/S) is defined as the ratio of the total water mass to the total solid material mass in the system, where the total solid material includes waste soil particles and cementitious materials. The detailed mixture design and experimental scheme are summarized in Table 2. The SR–GGBS–PG binder used in this study was adopted from previous work with an optimized mass ratio of SR:GGBS:PG = 10:81:9 [48]. In the final mix design, the total cementitious content was composed of 80% SR–GGBS–PG binder and 20% PC by mass, and the resulting proportion was expressed as SR:GGBS:PG:PC = 10:81:9:25 based on mass-ratio normalization.

For specimen preparation, WS and SGPC were first blended in a dry state to ensure uniform dispersion of the stabilizer. Subsequently, the calculated amount of water was gradually added, and the mixture was mixed using a JJ-5 planetary mixer at a rotational speed of 160 rpm until a homogeneous flowable slurry was obtained. The fresh slurry was then cast into cylindrical molds (39.1 mm in diameter and 80 mm in height) whose inner surfaces were coated with petroleum jelly to facilitate demolding. The molds were filled in three layers, and each layer was gently compacted before adding the next. After casting, the molds were placed on a vibrating table for approximately 40–60 s to remove entrapped air bubbles. The molds were sealed with plastic film to minimize moisture loss and transferred to a standard curing chamber maintained at 20 ± 2 °C with relative humidity higher than 95%. UCS specimens were placed under standard curing conditions after molding and allowed to stand for 24 h. Demolding was carefully carried out only when the specimens could maintain their basic integrity without obvious deformation, after which standard curing was continued. It should be noted that the demolding time in this study was primarily controlled based on the early shape stability of the specimens, rather than strictly corresponding to the initial setting time of the slurry determined by the Vicat method. Due to the presence of a soil particle skeleton and interparticle physical interlocking in the flowable stabilized soil system, the specimens can maintain a certain degree of structural integrity even before the cementitious system reaches the conventional initial setting state. Three parallel specimens were prepared for each mixture.

### 2.3. Nomenclature

To ensure consistency in terminology and notation throughout the manuscript, the nomenclature and abbreviations used in this study are summarized in Table 3.

### 2.4. Testing Methods

To evaluate the engineering performance and microstructural characteristics of the stabilized soil, a series of laboratory tests were conducted, including unconfined compressive strength, flowability, setting time, and microstructural characterization. For the macroscopic tests, at least three parallel specimens (n = 3) were prepared for each group, and the reported results represent the average values, with error bars indicating the standard deviation.

#### 2.4.1. Mechanical Properties

The mechanical strength of the stabilized soil was determined through the unconfined compressive strength (UCS) test in accordance with ASTM D2166/D2166M [49]. A computer-controlled compression testing machine was used to conduct the measurements. Before testing, the cylindrical specimens were carefully positioned between the loading plates to ensure proper alignment. Axial compression was then applied under displacement control with a constant loading rate of 1 mm/min until failure occurred. The peak load obtained during the test was used to calculate the unconfined compressive strength according to:

(1) $$P=\frac{F_{max}}{A}$$

where P represents the compressive strength, $F_{max}$ is the maximum applied load, and A denotes the cross-sectional area of the specimen.

#### 2.4.2. Working Properties

The workability of the freshly prepared stabilized slurry was evaluated using the flow table method in accordance with GB/T 2419-2005 [50]. The flow diameter was measured in two perpendicular directions, and the average value was used to characterize the flowability of the mixture.

The setting time of the stabilized slurry was determined using a Vicat apparatus in accordance with GB/T 1346-2011 [51]. The initial setting time was defined as the moment when the penetration depth of the standard needle reached 4 ± 1 mm above the bottom of the mold, while the final setting time was defined as the point at which no visible indentation could be observed on the specimen surface. This setting time test is used only to evaluate the relative evolution of early structural development under different mix proportions, and is not intended for direct comparison with standard setting times of ordinary cement systems.

#### 2.4.3. pH Measurement

The alkalinity of the stabilized system was evaluated by measuring the pH value of a soil–water suspension. Fragments collected from the interior of the UCS specimens after testing were air-dried and ground into fine powder. Approximately 10 g of the powder was mixed with 50 mL of deionized water to form a suspension. The mixture was stirred thoroughly and then allowed to stand until the reading became stable. A calibrated digital pH meter was used to record the pH value of the suspension.

#### 2.4.4. Microstructural Characterization

**Figure 6:**
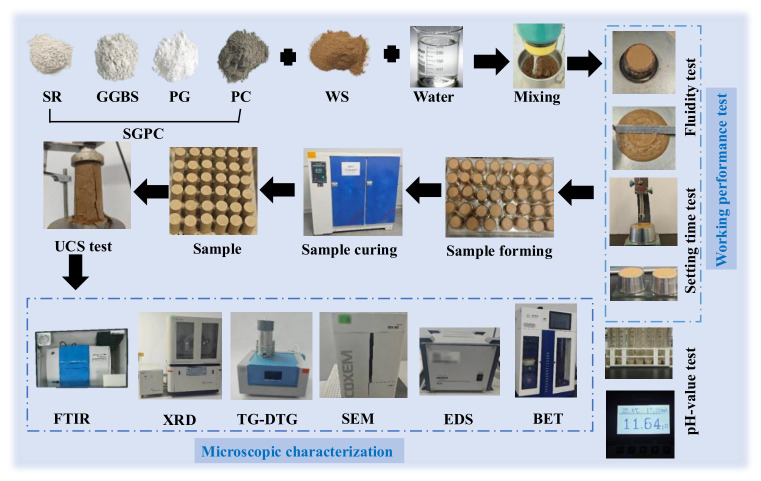
Flowchart of the experimental process for waste soil stabilization.

To further elucidate the reaction mechanism of the stabilized soil, a series of microstructural characterizations were conducted. Samples used for microscopic analyses were collected from the interior region of the UCS specimens to minimize boundary effects. The collected fragments were first immersed in isopropanol for 7 days to terminate ongoing hydration reactions and then dried at a moderate temperature until constant mass was achieved. Samples for FTIR, XRD, TG, and BET analyses were ground and passed through a 75 μm sieve, followed by sealing for subsequent testing. X-ray diffraction (XRD) analysis was performed using a TD-3500 diffractometer (Dandong Tongda Technology Co., Ltd., Dandong, China) to identify mineral phases. Fourier transform infrared spectroscopy (FTIR) was conducted using an FTIR-650S spectrometer (Gangdong Science & Technology Co., Ltd., Tianjin, China) to characterize the evolution of functional groups associated with hydration products. Thermogravimetric (TG) analysis was carried out using an HTG-2 thermal analyzer (Beijing Hengjiu Experimental Equipment Co., Ltd., Beijing, China) to investigate the thermal decomposition behavior of reaction products. The microstructure of the stabilized soil matrix was observed using a scanning electron microscope (SEM, EM-30, COXEM Co., Ltd., Daejeon, Republic of Korea), coupled with an energy-dispersive X-ray spectroscopy detector (EDS, XFlash 630 H, Bruker Corporation, Billerica, MA, USA), to analyze elemental distribution. Samples for SEM observation were prepared as blocks with dimensions of 5 mm × 5 mm × 5 mm, with freshly exposed surfaces coated with a conductive layer prior to imaging. Nitrogen adsorption measurements were conducted using a V-Sorb X800 specific surface area and pore size analyzer (Beijing Guoyi Precision Measurement Technology Co., Ltd., Beijing, China) to characterize pore structures, with pore size distribution analyzed based on BET theory and the HK model. A schematic of the overall experimental procedure is shown in Figure 6.

Figure 6 presents the overall experimental workflow for the stabilization of waste soil (WS). First, the WS, GGBS, PG, PC, SR, and water were mixed according to the designed proportions to prepare fresh flowable pastes, which were then tested for workability by measuring flowability and setting time. Next, the pastes were cast and cured to the specified age, followed by pH measurement and unconfined compressive strength (UCS) testing to characterize the evolution of the material’s alkalinity and mechanical performance. Finally, the broken samples from the UCS tests were subjected to FTIR, XRD, TG–DTG, SEM, EDS, and BET analyses to further elucidate the microscopic stabilization mechanisms of SGPC-stabilized WS.

## 3. Results and Discussion

### 3.1. Workability of FSS

**Figure 7:**
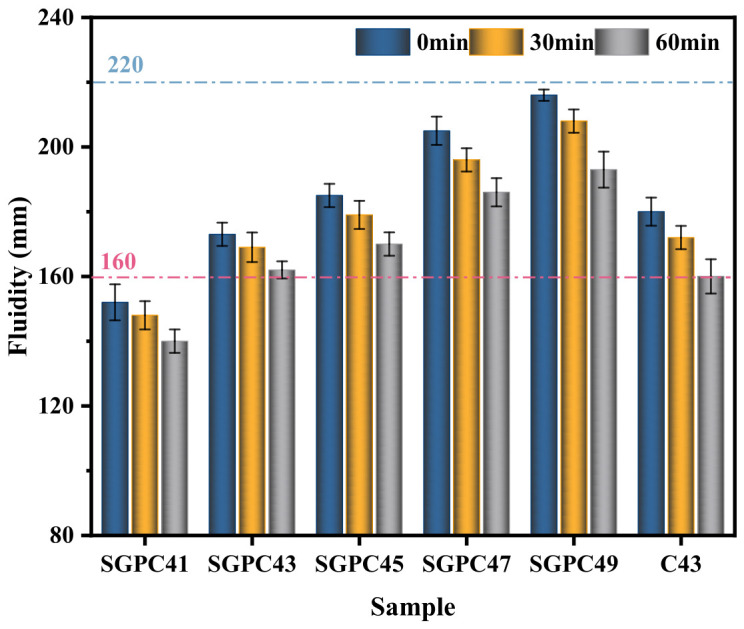
Fluidity loss over time for stabilized FSS.

Figure 7 illustrates the variation in flowability of the specimens measured at 0, 30, and 60 min under different water-to-solid ratios. As the water-to-solid ratio increased from 0.41 to 0.49, the initial flow diameter of the SGPC mixtures gradually increased from 152 mm to 216 mm. Specifically, the initial flow diameters of SGPC41, SGPC43, SGPC45, SGPC47, and SGPC49 were 152 mm, 173 mm, 185 mm, 205 mm, and 216 mm, respectively, showing a clear increasing trend with increasing water-to-solid ratio. According to the workability requirements of flowable materials in engineering applications and the relevant provisions of the Technical Specification for Application of Flowable Stabilized Soil in Construction Engineering (DB37/T 5345-2025), the flow spread of flowable stabilized soil mixtures is preferably controlled within the range of 160–220 mm to ensure adequate self-compacting ability and construction workability [52]. The initial flow diameter of SGPC41 was only 152 mm, which is lower than the commonly required range for flowable materials in engineering applications. This result indicates that the fluidity of the mixture is insufficient at a low water-to-solid ratio, which may limit its ability to fill confined spaces during construction. In contrast, when the water-to-solid ratio was relatively high, the flow diameters of SGPC47 and SGPC49 reached 205 mm and 216 mm, respectively, approaching the upper limit of the recommended range. Excessively high flowability may increase the risk of segregation and bleeding in the slurry system. Among the mixtures, SGPC43 exhibited an initial flow diameter of 173 mm, which was close to that of the cement-stabilized mixture C43 (180 mm) prepared with the same water-to-solid ratio. This indicates that the SGPC system can provide comparable flowability to the conventional cement-stabilized system under the same water condition. With increasing resting time, the flow diameter of all mixtures gradually decreased. This reduction can be attributed to the progressive hydration reactions of the cementitious materials. The formation of hydration products strengthens the interparticle structural network, resulting in increased viscosity of the slurry and consequently reduced flowability. Previous studies have reported that, in GGBS-based cementitious systems, an appropriate water-to-solid ratio can enhance the lubrication effect between particles and reduce the formation of flocculated structures, thereby improving the flow behavior of the slurry [53]. In addition, alkali-activated GGBS systems usually undergo an induction period during the early stage of reaction, allowing the material to retain relatively good flowability at early ages [54,55].

**Figure 8:**
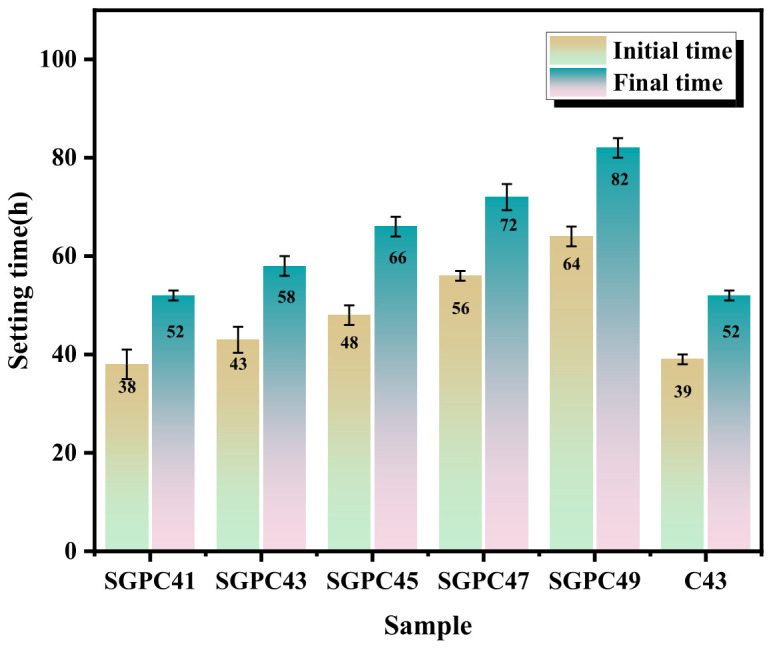
Setting time under different water–solid ratios.

Setting time is one of the key parameters for evaluating the transition of flowable solidified soil from a fluid state to a solidified body with structural strength, reflecting the process of skeleton formation and development within the system. As shown in Figure 8, both the initial and final setting times of the SGPC system gradually increase with increasing water–solid ratio. Specifically, when the water–solid ratio increases from 0.41, 0.43, 0.45, 0.47 to 0.49, the initial setting times are 38 h, 43 h, 48 h, 56 h, and 64 h, respectively, while the corresponding final setting times are 52 h, 58 h, 66 h, 72 h, and 82 h, indicating that the water–solid ratio has a significant regulatory effect on the setting process of the system. Under the same water–solid ratio of 0.43, the initial and final setting times of SGPC43 are 43 h and 58 h, respectively, whereas those of C43 are 39 h and 52 h, indicating that, compared with the conventional cement solidification system, the SGPC flowable solidified soil system exhibits a certain degree of setting retardation. This difference is mainly attributed to the fundamental differences in hydration reactions and structural formation mechanisms between the two systems. In the conventional cement solidification system, clinker minerals rapidly hydrate upon mixing with water, generating large amounts of C–S–H gel and Ca(OH)_2_, which quickly bind particles and form a continuous rigid skeleton structure, thereby accelerating the setting process. In the SGPC system, GGBS, as the main latent reactive component, gradually dissolves its glassy structure under alkaline conditions, releasing active ions such as Ca^2+^, SiO_4_^4−^, and [Al(OH)_4_]^−^. PC mainly provides a limited early Ca^2+^ source and hydration induction effect, SR provides an alkaline environment to promote the dissolution of GGBS, and PG participates in ionic balance and sulfate-related reaction processes within the system. Under the above multi-source synergistic effects, dissolved ions in the pore solution gradually form hydration products dominated by C–S–H and C–(A)–S–H through dissolution–migration–precipitation processes, while SO_4_^2−^ released from PG reacts with Ca^2+^ and aluminate components to form AFt, further regulating the early structure formation process. Compared with ordinary Portland cement systems, this multi-source reaction process has an overall slower rate, leading to a progressive development of hydration product formation and spatial connectivity [56], thereby delaying the formation of a continuous rigid skeleton and prolonging the transition time from a fluid state to a solidified state. In addition, with increasing water–solid ratio, the free water content in the system increases and the particle spacing enlarges, resulting in a decrease in effective ion concentration in the pore solution, which weakens interparticle interactions and delays nucleation and growth of hydration products, ultimately leading to prolonged initial and final setting times. Therefore, combining the results in Figure 7 and Figure 8, it can be concluded that at a water–solid ratio of 0.43, the SGPC system achieves a good balance between fluidity and setting time, ensuring a reasonable coordination between construction workability and operational time window. It should also be noted that relatively prolonged setting behavior is not necessarily unfavorable for flowable backfill applications, as an extended setting period may provide sufficient time for transportation, pumping, placement, and self-leveling during construction. Furthermore, the actual soil–binder composite system may develop earlier structural stability due to particle skeleton formation and interparticle interactions, which cannot be fully reflected by Vicat measurements alone.

### 3.2. Mechanical Properties of FSS

**Figure 9:**
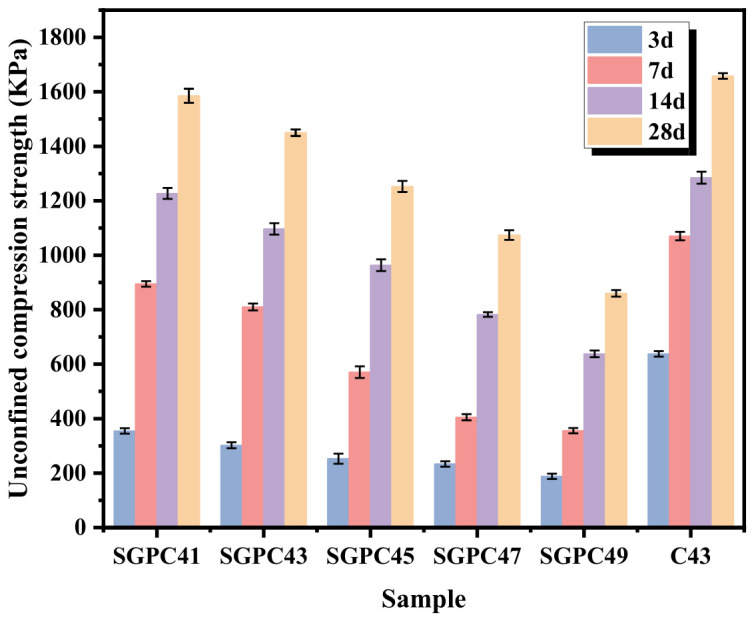
UCS values of solidified FSS under different water-solid ratios and curing ages.

Figure 9 illustrates the evolution of UCS of FSS under different curing ages. As shown, the UCS of all specimens significantly increased with prolonged curing time. Taking SGPC43 as an example, its UCS increased from 302.11 ± 10.92 kPa at 3 days to 810.11 ± 12.75 kPa at 7 days, further rose to 1096.55 ± 20.91 kPa at 14 days, and finally reached 1450.13 ± 11.99 kPa at 28 days. Compared with the value at 3 days, the UCS of SGPC43 at 7, 14, and 28 days increased by approximately 168.2%, 263.0%, and 379.8%, respectively, indicating continuous strength development over time. The other mix proportions exhibited a similar strength development trend to SGPC43. Specifically, the UCS of SGPC41 increased from 354.70 ± 9.99 kPa at 3 days to 1585.55 ± 25.69 kPa at 28 days, representing an increase of 347.1%; SGPC45 increased from 252.88 ± 18.59 kPa to 1252.63 ± 20.28 kPa, an increase of 395.4%; SGPC47 increased from 233.48 ± 10.04 kPa to 1074.18 ± 17.86 kPa, an increase of 360.1%; and SGPC49 increased from 187.98 ± 9.80 kPa to 860.15 ± 12.18 kPa, an increase of 357.6%. Among all mixtures, SGPC45 exhibited the largest relative increase (395.4%), whereas SGPC41 consistently showed the highest absolute strength. Overall, UCS at each curing age decreased with increasing water-to-solid ratio. To further evaluate data reliability, the results are presented as mean ± standard deviation (n = 3), and statistical analysis was performed. The results indicate that both curing age and mix proportion significantly affect UCS (*p* < 0.05), confirming that the observed differences are statistically significant rather than random fluctuations. In addition, the relatively small standard deviations for most specimens indicate good reproducibility and low data dispersion, suggesting that specimen preparation and testing procedures were stable and reliable. Meanwhile, noticeable differences in strength development were observed among specimens with different water-to-solid ratios. Overall, UCS decreased gradually with increasing water-to-solid ratio, with SGPC41 exhibiting the highest UCS at all curing ages, followed by SGPC43, SGPC45, SGPC47, and SGPC49. Previous studies have shown that in FSS systems, UCS generally decreases with increasing water content, as excessive water increases pore volume after hardening and reduces matrix compactness, which is detrimental to later strength development. This trend is consistent with Abrams’ law and findings from other researchers [57,58,59,60]. Therefore, the reduction in UCS at higher water-to-solid ratios in this study can be partly attributed to the formation of a looser internal structure after hardening. However, for FSS materials, the selection of an optimal mix proportion should consider not only mechanical performance but also workability requirements. Although SGPC41 exhibits slightly higher UCS, SGPC43 demonstrates superior flowability while maintaining relatively high strength, and is therefore identified as the mix proportion with the best overall performance. This indicates that the water-to-solid ratio not only governs strength development but also affects the balance between fresh and hardened properties of FSS systems.

Compared with the control group C43, SGPC43 exhibited a similar strength development trend. The UCS of C43 increased from 637.79 ± 10.33 kPa at 3 days to 1070.44 ± 15.55 kPa at 7 days, further increased to 1284.53 ± 22.01 kPa at 14 days, and finally reached 1658.26 ± 10.09 kPa at 28 days. Although the overall strength of C43 was slightly higher than that of SGPC43, the gap between the two gradually decreased with curing age. This is likely because the cement system continuously generates hydration products, forming a progressively denser cemented structure. Notably, the UCS of SGPC43 at 28 days reached 1450.13 kPa, approximately 87.45% of that of C43, indicating that the SGPC system prepared using industrial solid wastes can achieve mechanical properties comparable to those of traditional cement systems. Furthermore, SGPC43 exhibited a higher late-age strength gain rate, suggesting sustained reactivity and favorable long-term strength development potential.

It should be noted that although the unconfined compressive strength (UCS) at early curing ages, particularly at 7 days, is lower than the strength requirements commonly adopted for conventional structural soil stabilization applications, the SGPC system proposed in this study is primarily intended for flowable fill applications rather than high-bearing-capacity ground improvement. For flowable solidified soils used in self-compacting backfilling and trench-filling applications, workability and constructability are the primary design controlling parameters, while UCS is mainly used as an indicator of long-term stability. According to Clause 5.1.3 of the Compilation Explanation of the Technical Specification for Pre-mixed Flowable Solidified Soil (Draft for Comments), the recommended 28-day compressive strength for filling applications generally ranges from 300 to 2000 kPa [61]. The 28-day UCS of SGPC43 reached 1450.13 kPa, which falls within this recommended range. This indicates that, although the early-age UCS remains relatively low, the proposed SGPC system can satisfy the long-term performance requirements for non-structural flowable fill applications.

The most significant strength gain occurred during the early curing stage (3–14 days), indicating that hydration and activation reactions were highly active during this period. As curing time increased further, the strength gain rate gradually decreased, suggesting progressive densification and stabilization of the internal structure. Under alkaline conditions, GGBS continuously dissolves and reacts, producing C–(A)–S–H gel phases that fill pores and enhance interparticle bonding, thereby promoting late-age strength development. Similar phenomena have been widely reported in slag-based solidified soil systems [62]. This strength development mechanism can be interpreted from the perspective of pozzolanic reactivity of solid waste materials. The dissolution–reaction behavior is consistent with reaction pathways described in the evaluation framework for pozzolanic materials, and the reaction activity and formation characteristics of cementitious products in such systems have been systematically summarized in relevant evaluation frameworks [63]. Meanwhile, in solid waste-based fluidized solidified soil systems, GGBS, as a latent hydraulic material, gradually undergoes hydration reactions in the pore water environment of soil and, together with active components in the system, mainly forms cementitious products such as C–S–H and C–A–H, thereby significantly improving soil structure and mechanical properties Furthermore, recent studies on multi-source solid waste solidified soil indicate that synergistic interactions among different industrial solid wastes can promote continuous formation of hydration products and structural densification, and the microstructural evolution process is closely correlated with strength development [64]. The mass loss behavior observed in TG-DTG analysis also indirectly supports the progressive accumulation of hydration products with curing age, thereby validating the proposed strength development mechanism.

### 3.3. Chemical Environment Analysis

**Figure 10:**
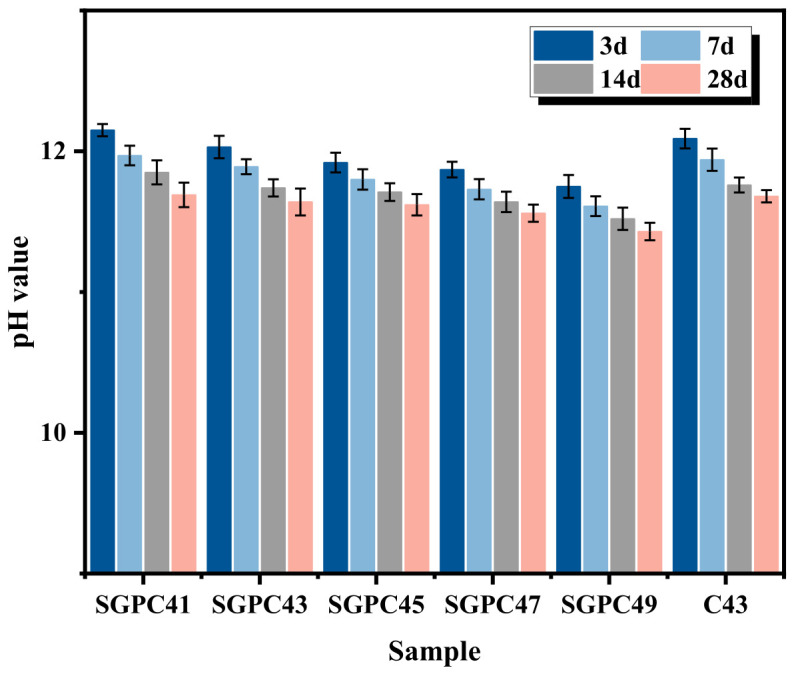
pH values of solidified FSS under different water–solid ratios and curing ages.

Figure 10 shows the variation in pH values of stabilized soils with different mix proportions at various curing ages. Overall, all specimens maintained a highly alkaline environment during the curing period, with pH values ranging approximately from 11.4 to 12.2. This indicates that the pore solution system is strongly alkaline rather than a neutral hydration environment. In this study, pH is used to characterize the chemical environment of the pore solution and to analyze its influence on dissolution and hydration processes.

With increasing curing age, the pH values of all specimens show a gradual decreasing trend. For example, the pH of SGPC43 decreased from approximately 12.0 at 3 days to 11.6 at 28 days. Similarly, the other mixtures exhibit the same downward trend: SGPC41 decreased from 12.15 to 11.69, SGPC45 from 11.92 to 11.62, SGPC47 from 11.87 to 11.56, and SGPC49 from 11.75 to 11.43. The total reduction in pH within 28 days ranges from 0.30 to 0.46, while all values remain above 11.4, indicating that a strongly alkaline environment is maintained. Overall, as the water-to-solid ratio increases from SGPC41 to SGPC49, the pH value at the same curing age decreases sequentially, which is consistent with the dilution effect of alkaline ions. This trend is mainly attributed to the continuous hydration reaction. Under a highly alkaline environment, the glassy phase of GGBS gradually dissolves, releasing ions such as Ca^2+^, SiO_4_^4−^, and Al(OH)_4_^−^. These ions participate in the formation of C–(A)–S–H gel. During this process, OH^−^ is consumed, leading to a slight reduction in alkalinity with curing age. Therefore, pH primarily reflects the consumption of OH^−^ and the reaction progress, rather than serving as a quantitative indicator of material reactivity.

In addition, the water-to-solid ratio influences the alkalinity of the system. As the water-to-solid ratio increases from 0.41 to 0.49, the pH value slightly decreases. This is mainly due to the dilution of alkaline ions in the pore solution. However, all specimens still maintain a strongly alkaline condition throughout the curing period (pH > 11), which ensures the continuous dissolution and hydration of GGBS. The stable high-alkaline environment is beneficial for slag activation and promotes the formation of cementitious products. Further analysis of variability shows that the standard deviation of all mixtures at different ages ranges from 0.0436 to 0.0954, and the coefficient of variation is below 1.0%, indicating good repeatability of the parallel specimens. The standard deviations of SGPC41 and SGPC43 slightly increase with curing age (from 0.0436 to 0.0872, and from 0.0794 to 0.0954, respectively), which may reflect increased heterogeneity of hydration at later stages. One-way ANOVA results indicate that the 28-day pH values differ significantly among different mix proportions (*p* < 0.01), demonstrating that the increase in water-to-solid ratio has a statistically significant dilution effect on pH. This statistical result also provides supplementary evidence for explaining the variability of UCS shown in Figure 9.

Previous studies have shown that a highly alkaline environment facilitates the dissolution of the slag glass phase and the formation of a stable C–(A)–S–H structure [65]. In addition, the control group C43 consistently exhibits high pH values at all curing ages, mainly due to the generation of Ca(OH)_2_ from cement hydration, which helps maintain system alkalinity [66].

Overall, the sustained highly alkaline environment provides essential conditions for the dissolution and hydration of GGBS, thereby promoting the formation of C–(A)–S–H and further densifying the structure of the stabilized soil. This finding is consistent with the increasing trend of UCS shown in Figure 9. Therefore, pH should be regarded as an indirect indicator reflecting the chemical environment of the pore solution and reaction progress, which is used to assist in interpreting strength development, rather than a direct quantitative measure of material reactivity.

### 3.4. Hydration Products and Structural Evolution of FSS

To further elucidate the influence of the water-to-solid ratio on the hydration reactions and structural evolution of SGPC-based flowable stabilized soil, FTIR and XRD analyses were conducted to investigate the variations in functional groups and the evolution of crystalline phases in different systems. These results were then interpreted in conjunction with the macroscopic workability and mechanical performance to provide a comprehensive discussion of the internal structural changes of the systems.

**Figure 11:**
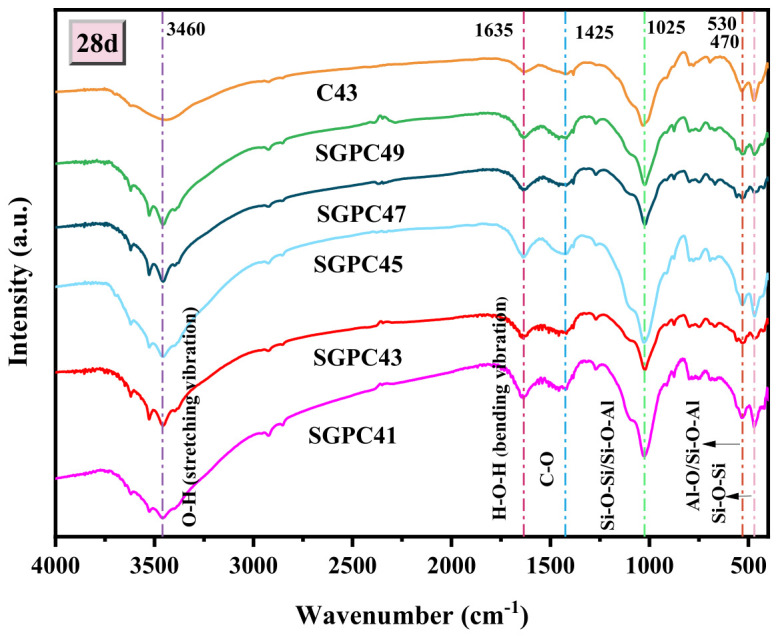
FTIR spectra of FSS under different water–solid ratios.

Figure 11 shows the FTIR spectra of the FSS samples at a curing age of 28 days. Overall, the characteristic peak positions of all samples are basically consistent, and no new absorption peaks or significant peak shifts were observed, indicating that the types of functional groups in the hydration products formed under different water-to-solid ratios are similar, and that the water-to-solid ratio has no significant effect on the chemical environment of the hydration products. FTIR analysis primarily provides qualitative information on functional groups and is used in this study as a supplementary characterization method, interpreted in combination with other experimental results to further understand hydration features. The enhancement of characteristic absorption peaks and minor peak shifts indicate continuous formation and structural polymerization of hydration gels within the system, and a denser gel network formed under lower water-to-solid ratios contributes to improved internal structural compactness, thereby promoting the development of unconfined compressive strength. In the high wavenumber region, a broad absorption band appears around 3460 cm^−1^, typically attributed to O–H stretching vibrations of bound and structural water, indicating the presence of hydrated phases such as C–S–H-type products [67]; a weak peak at approximately 1640 cm^−1^ corresponds to the bending vibration of H–O–H in adsorbed or physically bound water, suggesting that residual water remains in the system after 28 days of curing [68]. In the mid-wavenumber region, the absorption band at 1425 cm^−1^ is mainly associated with the asymmetric stretching vibration of C–O bonds in carbonate groups, indicating slight carbonation due to interaction with environmental CO_2_ and possible formation of carbonate-containing phases [69], whereas a relatively strong peak at around 1025 cm^−1^ is attributed to the asymmetric stretching of Si–O–Si or Si–O–Al (Si–O–T, T = Si or Al) bonds, characteristic of aluminosilicate gel structures such as C–(A)–S–H-type gels [70,71], which are typically formed in alkali-activated or slag-based systems through dissolution and polymerization of aluminosilicate precursors. In the low wavenumber region, a peak near 530 cm^−1^ is assigned to vibrations of Si–O–Al or Al–O bonds in AlO_4_ tetrahedra, indicating aluminum incorporation into the gel network [72], and a peak at approximately 470 cm^−1^ corresponds to the bending vibrations of Si–O–Si bonds in SiO_4_ tetrahedra, reflecting the presence of silicate tetrahedral units in the hydrated matrix [73].

**Figure 12:**
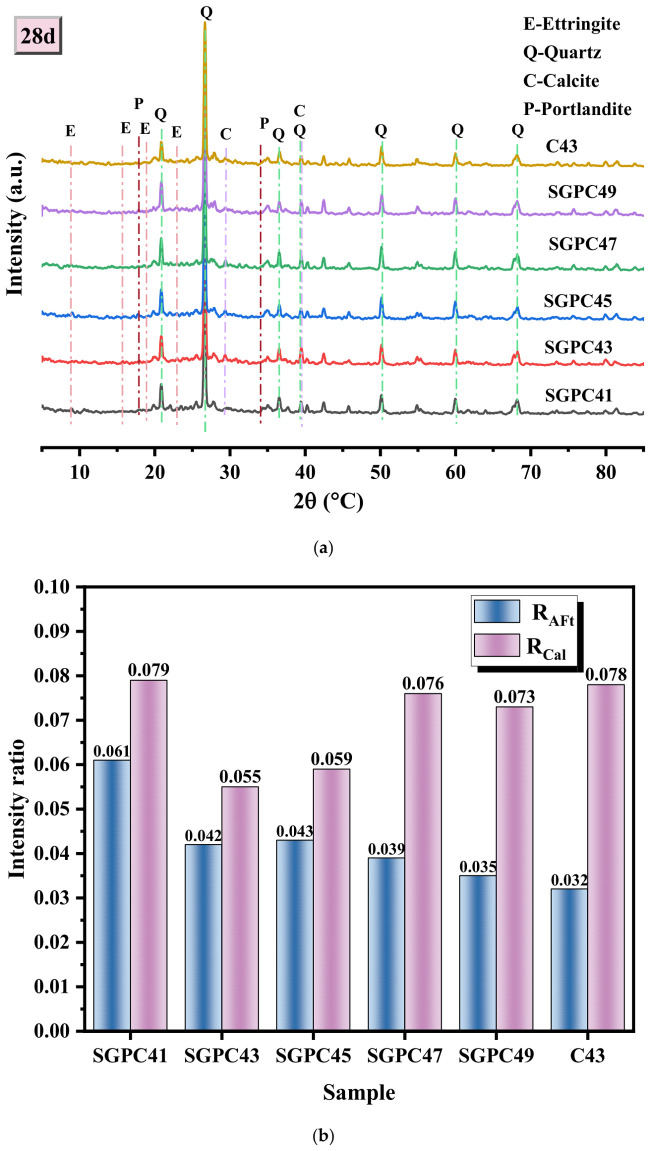
(**a**) XRD patterns under different water-solid ratios; (**b**) Relative variation of crystalline phases based on XRD characteristic peak intensity ratios.

Figure 12 presents the XRD patterns of SGPC-stabilized soils with different water-to-binder ratios and the pure cement control group (C43) after 28 days of curing. Characteristic diffraction peaks of AFt, quartz, calcite, and CH are observed in all samples, indicating that the primary hydration products are generally consistent among the different systems. AFt is mainly formed through the reaction between sulfate ions released from PG and the aluminate phases in cement and GGBS, which is a typical hydration feature of the GGBS–PG co-activated system. Previous studies have reported that in slag–phosphogypsum composite cementitious systems, PG can provide sufficient sulfate ions to promote the formation of AFt and accelerate the generation of C–(A)–S–H gel, thereby improving the structural compactness and mechanical performance of the matrix [74,75].

By comparing samples with different water-to-binder ratios, it can be observed that the diffraction peak intensity of AFt gradually decreases with increasing water-to-binder ratio. For example, the peak at approximately 2θ ≈ 9.1° is the most pronounced in SGPC41, whereas the peak intensity in SGPC49 is significantly reduced. This phenomenon may be attributed to the dilution of ionic concentration under higher water-to-binder ratios, which weakens the precipitation and crystallization of AFt. XRD results further indicate that changes in the water-to-solid ratio affect the degree of hydration reaction and the phase composition evolution of the system, and these changes show good consistency with the macroscopic strength development. In addition, a broad amorphous hump appears in the range of 2θ = 20–35°, which is generally attributed to the poorly crystalline C–(A)–S–H gel. Previous studies have indicated that AFt and C–(A)–S–H gel are the dominant hydration products in PG–GGBS-based cementitious systems, and their interwoven microstructure contributes to the formation of a dense matrix, thereby significantly enhancing the strength and durability of the stabilized material [76,77]. Therefore, a lower water-to-binder ratio is beneficial for the formation of hydration products and the development of a denser microstructure, whereas a higher water-to-binder ratio may lead to a relatively loose internal structure of the stabilized soil.

In the present study, a semi-quantitative analysis of XRD data was performed to characterize the relative variation of crystalline phase composition among different samples. Considering the relatively high proportion of amorphous hydration products in the system, full-pattern Rietveld refinement was not applied. Instead, the relative evaluation was based on the intensity ratios of characteristic diffraction peaks. Quartz (SiO_2_, 2θ = 26.6°) was used as the internal reference phase, while the characteristic peaks of ettringite (AFt, 2θ ≈ 9.1°) and calcite (CaCO_3_, 2θ ≈ 29.4°) were selected. The relative intensity ratios were calculated as follows:

(2) $$R_{AFt}=\frac{I_{AFt}}{I_{Qtz}}$$

(3) $$R_{Cal}=\frac{I_{Cal}}{I_{Qtz}}$$

where $I_{AFt}$, $I_{Cal}$, and $I_{Qtz}$ represent the diffraction peak intensities of ettringite, calcite, and quartz, respectively. This approach reflects the relative variation trends of the crystalline phases rather than their absolute contents. The results indicate that $R_{AFt}$ varies between 0.032 and 0.061, whereas $R_{Cal}$ ranges from 0.055 to 0.079. Among the samples, SGPC41 exhibits the highest $R_{AFt}$ value, which generally decreases with increasing water-to-solid ratio, reaching the lowest value in SGPC49. This trend suggests that higher water content may reduce the relative formation of ettringite. In contrast, the variation in $R_{Cal}$ is comparatively small, indicating that the response of calcite to changes in mix proportion is less pronounced than that of ettringite. Overall, the semi-quantitative XRD results demonstrate that different mix proportions primarily affect the formation of ettringite, while their influence on calcite is relatively limited.

### 3.5. Hydration Product Characteristics of FSS

**Figure 13:**
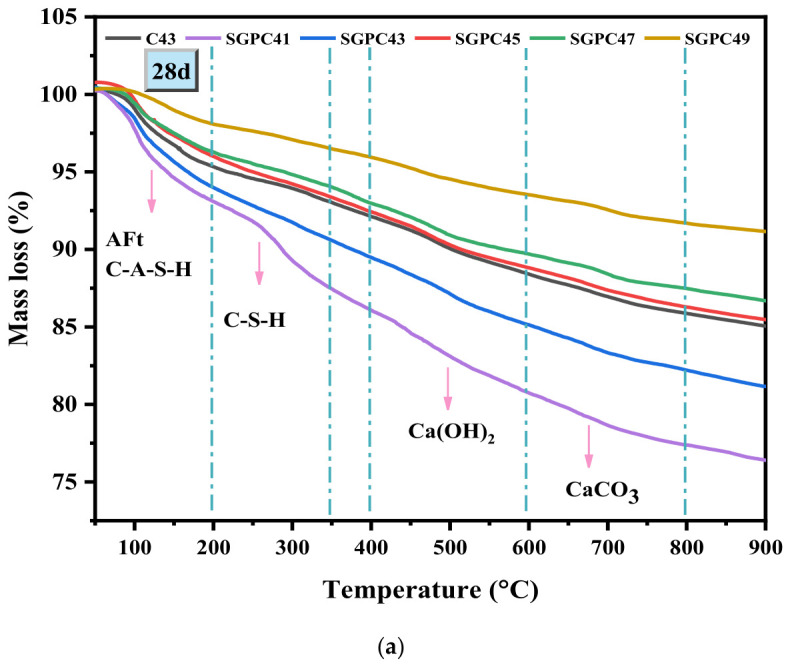

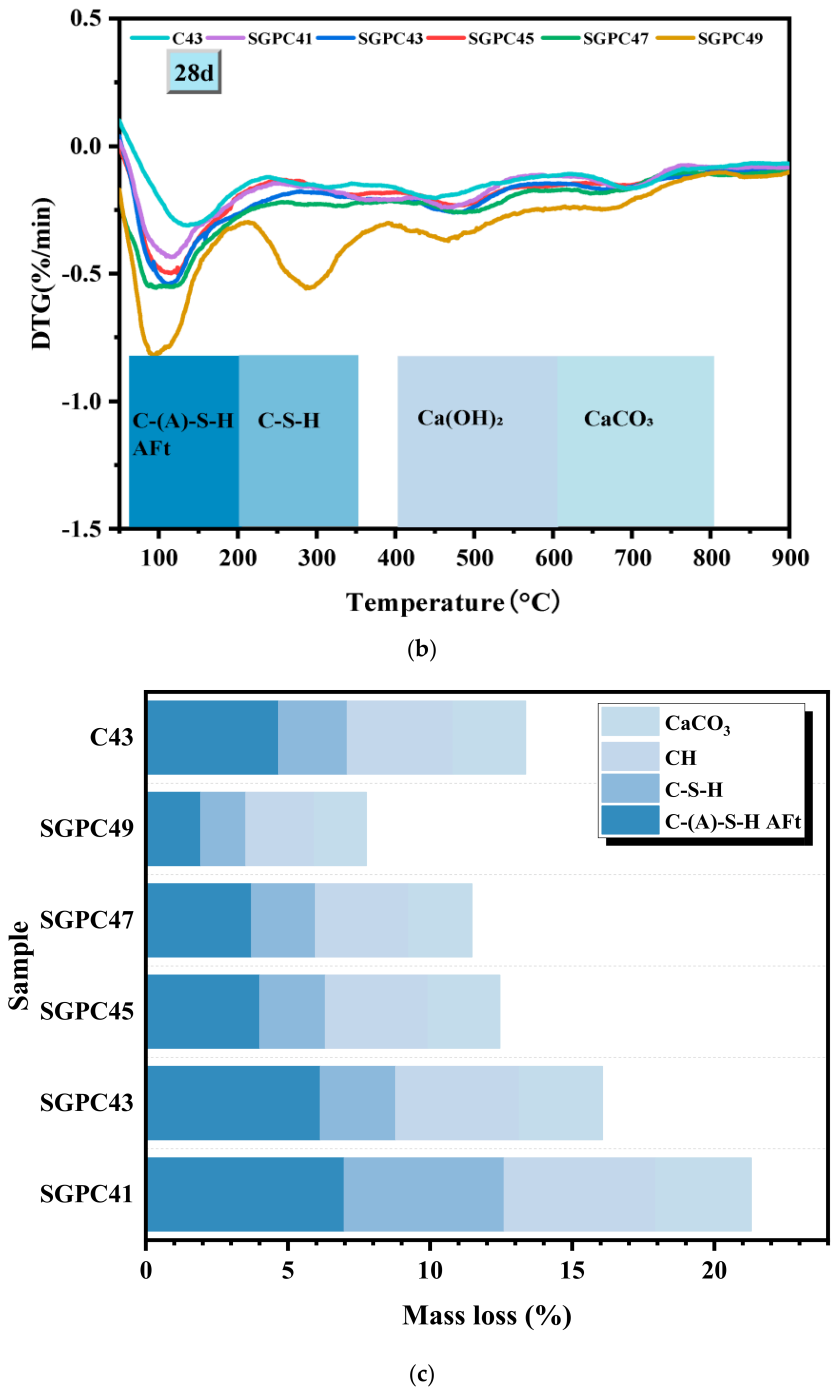
TG-DTG curves of stabilized FSS under different water–solid ratios: (**a**) TG curves; (**b**) DTG curves; (**c**) the mass loss within different temperature intervals.

TG–DTG is applied to quantify the formation of main hydration products such as AFt, C–(A)–S–H gel, and CH. To further elucidate the hydration products of SGPC-stabilized soils with different water-to-binder ratios and their relationship with macroscopic properties, TG–DTG was performed on specimens cured for 28 days. The results are presented in Figure 13, where Figure 13a shows the TG curves, Figure 13b shows the DTG curves, and Figure 13c summarizes the mass loss within different temperature intervals. The TG results were used to estimate the relative contents of hydration products based on typical decomposition temperature intervals, thereby reflecting the hydration evolution trend rather than absolute quantities. As shown in the TG curves, the mass of all samples gradually decreased with increasing temperature, although the extent of mass loss varied significantly with the water–binder ratio. The mass loss within 100–200 °C is mainly attributed to the dehydration of C–(A)–S–H gel and AFt [78,79]. In this temperature range, the mass losses of SGPC41, SGPC43, SGPC45, SGPC47, and SGPC49 were 6.98%, 6.14%, 4.01%, 3.72%, and 1.93%, respectively, showing a decreasing trend with increasing water–binder ratio. This indicates that a lower water–binder ratio promotes the formation of AFt and C–(A)–S–H gel, which is consistent with the stronger AFt diffraction peak observed in the XRD results for SGPC41.

Continuous mass loss in the 200–350 °C range corresponds to the further dehydration of C–S–H gel [80], suggesting that the gel phase plays an important role in forming the binding structure. A pronounced mass loss peak appeared within 400–600 °C, which is associated with the dehydroxylation of CH [81]. The CH mass losses of SGPC41, SGPC43, SGPC45, SGPC47, and SGPC49 were 5.33%, 4.35%, 3.61%, 3.28%, and 2.41%, respectively, also decreasing with increasing water–binder ratio. This may be attributed to the higher hydration degree of cement at lower water–binder ratios, producing more initial CH, while the pozzolanic reaction of slag consumes CH and generates additional C–(A)–S–H gel, thereby enhancing microstructural densification. Moreover, the mass loss observed in the 600–800 °C range is mainly related to the decomposition of CaCO_3_, which may result from slight carbonation during curing [82]. Overall, SGPC41 exhibited the highest total mass loss at 800 °C (20.86%), whereas SGPC49 showed the lowest value (7.76%), indicating that lower water–binder ratios lead to greater hydration product formation. Therefore, TG–DTG results demonstrate that lower water–binder ratios promote the formation of AFt and C–(A)–S–H gel and contribute to a denser microstructure, whereas higher water–binder ratios reduce the degree of hydration and hydration product formation, leading to strength deterioration in stabilized soils. The interpretation of the decomposition temperature ranges of hydration products is consistent with recent thermogravimetric studies on cement-based materials.

### 3.6. Microstructural Evolution and Pore Structure Characteristics of FSS

**Figure 14:**
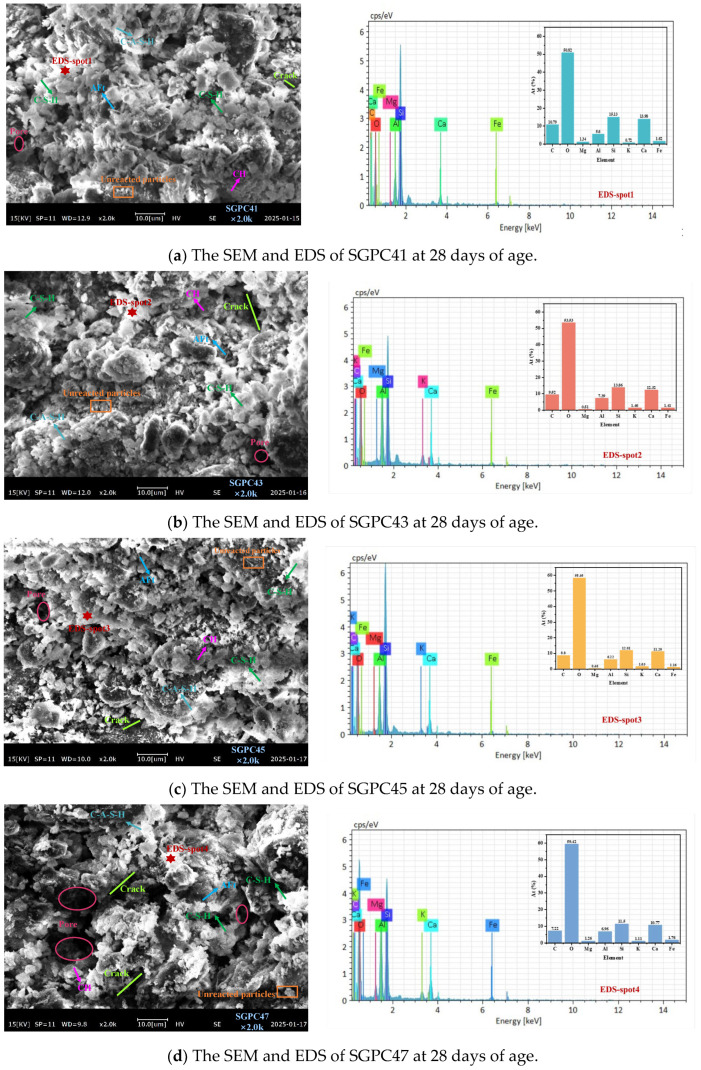

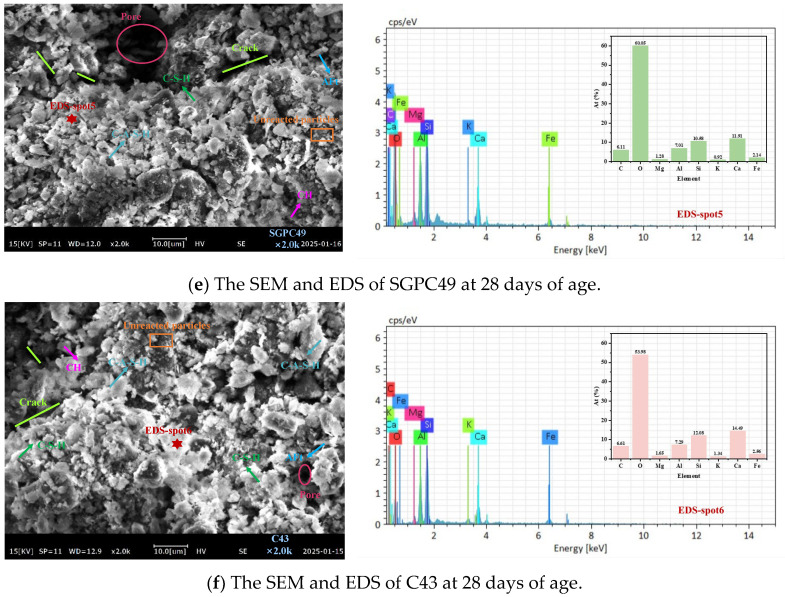
SEM and EDS results of different water–solid ratios.

SEM–EDS and BET were used to characterize the microstructure and pore evolution of SGPC-stabilized FSS. SEM–EDS analysis was conducted on 28-day cured specimens with different water-to-binder ratios (SGPC41–SGPC49) and the control sample C43, as shown in Figure 14. SGPC41–SGPC49 correspond to Spot1–Spot5, and C43 corresponds to Spot6. Overall, although similar features such as unreacted particles, pores, and microcrack-like structures are observed in all specimens, their frequency, spatial distribution, and connectivity differ significantly, indicating a systematic evolution from a dense and continuous matrix to a progressively loose and heterogeneous structure with increasing water-to-binder ratio. SGPC41 (Spot1, W/S = 0.41) exhibits the most compact microstructure among all specimens. A continuous and interwoven gel network is observed, which effectively fills interparticle voids. Only a limited number of unreacted particles are present, and their boundaries are relatively indistinct, indicating a higher degree of hydration. EDS results show that this region is mainly composed of O, Si, Al, and Ca elements, suggesting the formation of C–(A)–S–H type gel phases. In SGPC43 (Spot2, W/S = 0.43) and SGPC45 (Spot3, W/S = 0.45), hydration products remain abundant; however, the continuity of the gel network begins to decrease. Compared with SGPC41, pores appear more frequently and particle boundaries become clearer, indicating a gradual reduction in structural compactness with increasing water content. When the water-to-binder ratio further increases to SGPC47 (Spot4, W/S = 0.47) and SGPC49 (Spot5, W/S = 0.49), the microstructure becomes significantly more heterogeneous. The gel phase becomes discontinuous, while pore connectivity and microstructural disorder increase markedly. In addition, localized microcrack-like features are more frequently observed, indicating a weakened bonding framework and enhanced development of connected pore channels. In contrast, the control sample C43 (Spot6, W/S = 0.43) exhibits sheet-like or agglomerated hydration products. EDS results indicate a Ca–Si–O dominated composition, suggesting that C–S–H is the main hydration product. However, its spatial continuity and packing density are lower compared with SGPC41. Previous studies have shown that the formation and spatial distribution of C–(A)–S–H or C–S–H gels play a crucial role in determining the structural compactness and mechanical performance of cement-based materials, whereas a higher water-to-binder ratio generally increases porosity and weakens the continuity of the gel network [83,84]. Moreover, Li et al. reported that in systems containing reactive components such as slag, the formation of C–(A)–S–H gel can effectively refine the pore structure and enhance the overall compactness of the material [85]. Importantly, the SEM results indicate that the key difference among specimens lies not in the presence of similar hydration products or defects, but in the degree of gel network connectivity and pore structure development, which governs the transition from a dense to a loose microstructure. The observed microstructural evolution is consistent with previous studies on solid waste-based cementitious systems, which reported that hydration products derived from industrial by-products contribute to pore refinement and matrix densification, thereby improving mechanical performance [86,87,88]. These studies further support that changes in pore structure and gel continuity play a key role in governing the macroscopic behavior of stabilized soils. As the water-to-binder ratio increases, the deterioration in macroscopic mechanical performance can therefore be attributed to the progressive loss of gel network continuity and the enhanced development of connected pore structures.

**Figure 15:**
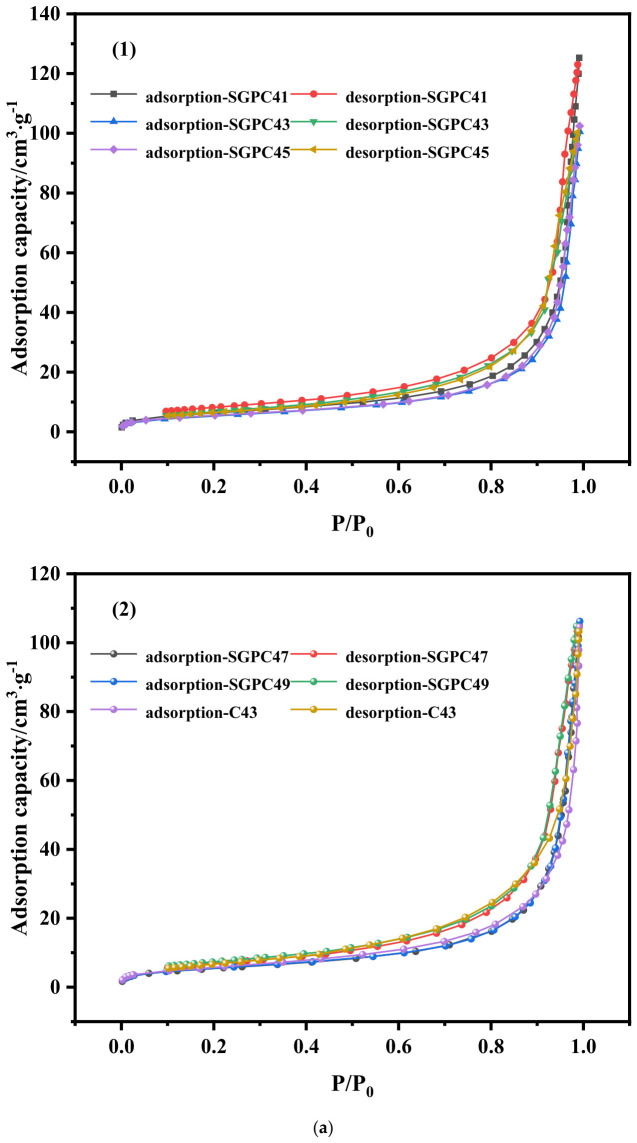

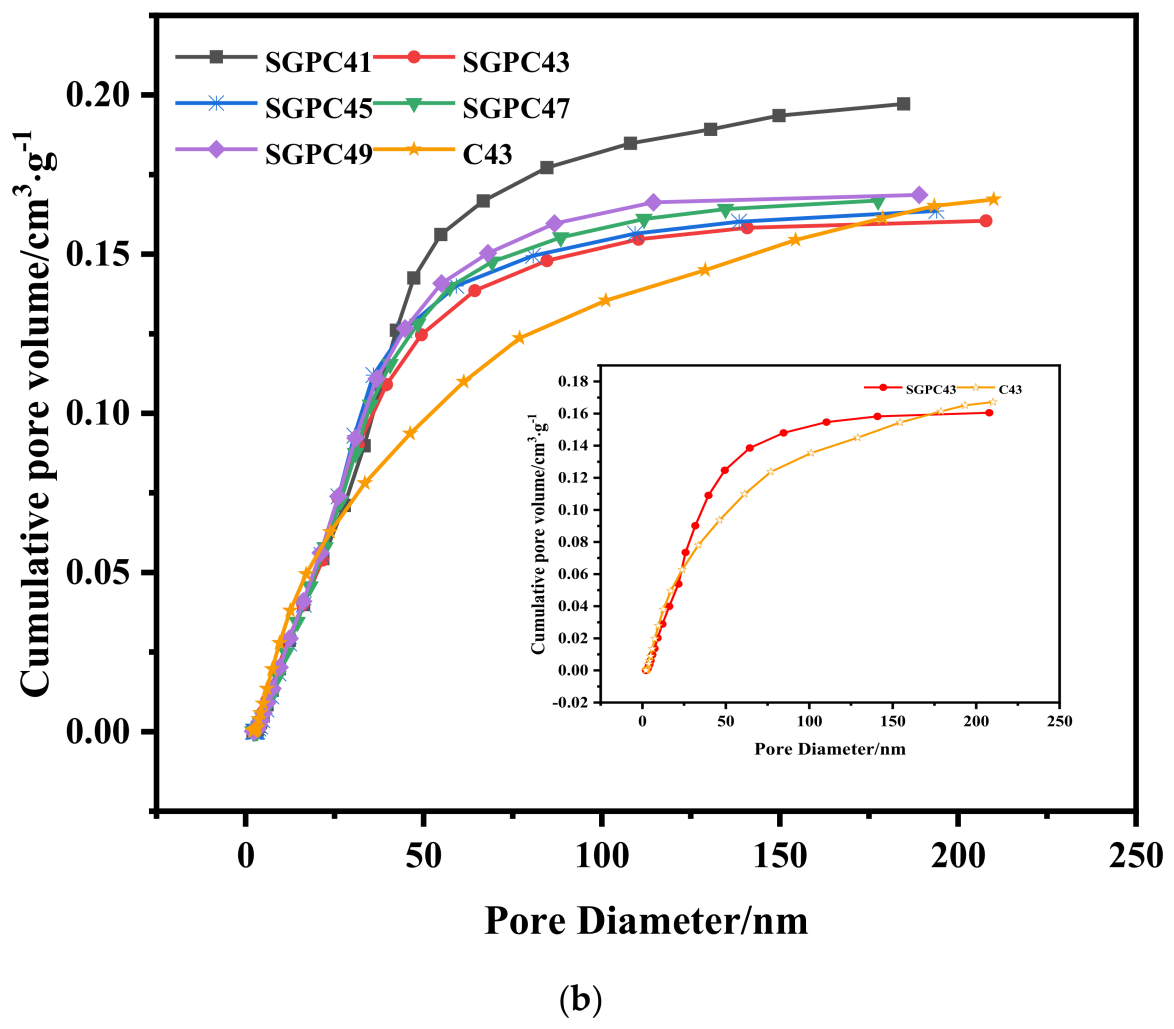
BET analysis of stabilized FSS under different water–solid ratios: (**a**) Isotherm diagram of adsorption and desorption: (**1**) SGPC41–SGPC45; (**2**) SGPC47–SGPC49 and C43; (**b**) cumulative pore volume distribution.

Figure 15 presents the nitrogen adsorption–desorption isotherms (Figure 15a) and cumulative pore volume distribution curves (Figure 15b) of SGPC specimens with different water-to-solid ratios after 28 days of curing, together with the control group C43. According to the classification of the International Union of Pure and Applied Chemistry (IUPAC), all samples exhibit typical type IV adsorption–desorption isotherms, accompanied by pronounced H3 hysteresis loops within the relative pressure range of P/P_0_ = 0.4–1.0. Type IV isotherms generally indicate the presence of mesoporous structures (2–50 nm), while H3 hysteresis loops are commonly associated with slit-shaped pores formed by the stacking of plate-like particles. Although partial overlap is observed among the isotherm curves, the differences in adsorption behavior remain clearly distinguishable, indicating distinct pore development levels induced by different water-to-solid ratios. This observation is consistent with the SEM results, where gel products and unreacted particles interconnect to form layered microstructures. These results suggest that the SGPC-stabilized system develops a hierarchical pore structure dominated by a gel skeleton. Previous studies have reported that in slag-containing cementitious systems, the formation of C–(A)–S–H gel can significantly modify the pore structure distribution and refine the mesoporous network, thereby improving the compactness and mechanical properties of the material [89]. In terms of nitrogen adsorption capacity, SGPC41 (W/S = 0.41) exhibits the lowest adsorption amount among all specimens, with a maximum adsorption capacity of approximately 0.14 cm^3^·g^−1^. As the water-to-solid ratio increases to 0.49 (SGPC49), the adsorption capacity increases significantly, with an overall increment exceeding 30%. This clearly indicates a progressive increase in accessible pore space with increasing water-to-solid ratio, highlighting a systematic evolution of pore structure. According to BET theory, nitrogen adsorption reflects the accessible pore surface area and pore volume within the material. A lower adsorption capacity generally indicates fewer accessible pores and a denser pore structure. The lowest adsorption capacity observed in SGPC41 suggests that hydration reactions are more extensive under a lower water-to-solid ratio, generating abundant C–(A)–S–H and C–S–H gels that effectively fill interparticle voids and reduce the pore space accessible to nitrogen molecules. This result is consistent with the dense gel network structure observed in the SEM analysis.

The cumulative pore volume distribution further reveals the influence of the water-to-solid ratio on pore structure evolution. SGPC41 shows the lowest cumulative pore volume across all pore size ranges, with a final cumulative pore volume of approximately 0.10 cm^3^·g^−1^, whereas SGPC49 reaches about 0.16 cm^3^·g^−1^. In terms of pore size distribution, most pores in all specimens fall within the mesopore range (2–50 nm), and the pore volume in the 20–50 nm range increases most significantly with increasing water-to-solid ratio. This trend further confirms that pore coarsening is concentrated in the mesoporous region, which is the most sensitive range to water content variation. As the water-to-solid ratio increases, more free water is present in the system, which subsequently evaporates during the hardening process and leaves behind more capillary pores, leading to a more porous microstructure and weakening the continuity of the gel network. It should be noted that although SGPC41 exhibits the lowest pore volume and the most compact pore structure, an excessively low water-to-solid ratio significantly reduces the flowability of the slurry, which is unfavorable for meeting the workability requirements of flowable fill materials. In contrast, SGPC43 maintains a relatively dense pore structure with only a slightly higher pore volume than SGPC41 while still being significantly lower than those of the higher water-to-solid ratio specimens (SGPC45–SGPC49). From a combined pore structure–mechanical perspective, the evolution of nitrogen adsorption and pore size distribution provides a direct microstructural explanation for the observed differences in macroscopic performance. Similar pore structure optimization behavior has also been reported in slag-based cementitious systems [90]. Therefore, within the investigated range of water-to-solid ratios, SGPC43 achieves a better balance between pore structure compactness and workability, resulting in the most favorable overall engineering performance.

### 3.7. Evaluation of Economic and Environmental Benefits

From the perspective of sustainable development, the SGPC stabilization system demonstrates significant environmental and economic advantages. Since the binder compositions of SGPC41–SGPC49 remained identical and only the water-to-solid ratio varied, their embodied carbon emissions and material costs were considered identical in this study. Therefore, SGPC43, which exhibited the best overall mechanical and microstructural performance, was selected as the representative SGPC system for environmental and economic evaluation and compared with the pure cement control group C43 under the same water-to-solid ratio condition. SGPC43 consists of industrial solid wastes including SR, GGBS, and phosphogypsum PG, with only 20% PC, resulting in an 80% solid waste utilization rate, whereas C43 is fully based on PC. The utilization of industrial by-products as cementitious materials has been widely recognized as an effective strategy for reducing environmental burdens and improving resource efficiency in cement-based systems [91,92,93,94,95].

To quantitatively evaluate environmental and economic performance, a simplified cradle-to-gate life cycle assessment (LCA) method was adopted, based on the principles of ISO 14040/14044 [96,97]. The system boundary includes only raw material production, while transportation, construction, maintenance, and service-life stages are not considered. The embodied carbon emission and material cost of the binder system were calculated using the following equations:

(4) $$C=\sum_{i=1}^{n}m_{i}\cdot EF_{i}$$

(5) $$Cost=\sum_{i=1}^{n}m_{i}\cdot P_{i}$$

where C is the total embodied carbon emission (kg CO_2_-e/t), Cost is the total material cost (USD/t), $m_{i}$ is the mass fraction of each component, $EF_{i}$ is the embodied carbon emission factor, and $P_{i}$ is the corresponding unit market price.

The embodied carbon factor of ordinary Portland cement (PC) was taken as 0.93 kg CO_2_-e/kg based on the ICE database [91], while that of ground granulated blast furnace slag (GGBS) was taken as 0.07 kg CO_2_-e/kg according to the representative literature [92]. For PG and SR, due to the lack of unified database values, conservative literature-based ranges were adopted (PG: 0.01–0.03 kg CO_2_-e/kg; SR: 0.01–0.05 kg CO_2_-e/kg), and representative values of 0.02 kg CO_2_-e/kg and 0.03 kg CO_2_-e/kg were used for calculation, respectively [93,94,95]. Material prices were obtained from regional market quotations and industrial price reports. The adopted parameters are summarized in Table 4, and the calculated results are presented in Table 5. Compared with the traditional C43 system, SGPC43 reduces embodied carbon emissions from 930 kg CO_2_-e/t to 235 kg CO_2_-e/t, corresponding to a reduction of approximately 74.7%. Meanwhile, the material cost decreases from 110 USD/t to 53 USD/t, representing a reduction of approximately 51.8%. The significant environmental benefit is mainly attributed to the replacement of high-carbon Portland cement with large amounts of industrial solid wastes with low embodied carbon intensity, while the economic benefit arises from the lower market prices of GGBS, PG, and SR. To evaluate the robustness of the results, a simplified uncertainty analysis was conducted by varying key parameters (carbon emission factors and material prices) within ±20%. The results indicate that the carbon reduction ratio remains within 70% ± 5%, while the cost reduction remains within 50% ± 5%, confirming that the conclusions are not sensitive to reasonable parameter variations and exhibit good stability. Overall, although based on a simplified cradle-to-gate assessment, the results clearly demonstrate that the SGPC system achieves a favorable balance among environmental benefit, economic efficiency, and engineering performance, indicating strong potential for sustainable flowable fill applications.

### 3.8. Mechanism Analysis

**Figure 16:**
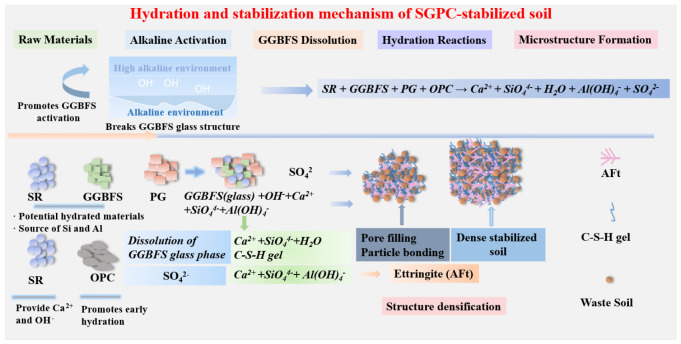
Schematic diagram illustrating the hydration and FSS immobilization mechanisms of SGPC.

Figure 16 systematically illustrates the hydration and solidification mechanism of SGPC (solid waste-based cementitious stabilizer) in stabilized soil. The system is mainly composed of SR, GGBS, PG, and PC, where SR and PC jointly provide an alkaline environment, GGBS serves as the primary aluminosilicate precursor, and PG supplies SO_4_^2−^. Together, these components form an alkali–sulfate synergistic activation system. The components do not react independently; instead, they are coupled through a continuous dissolution–hydration–precipitation process, which governs the formation of the final cemented structure. In the initial hydration stage, PC first undergoes rapid hydration with water, producing C–S–H gel while releasing Ca^2+^ and OH^−^, accompanied by the formation of CH (Ca(OH)_2_), which rapidly increases the alkalinity of the pore solution. Meanwhile, the dissolution of SR further contributes alkaline ions, maintaining a persistently high pH in the system. This strong alkaline environment not only promotes the breaking of Si–O–Si and Al–O–Si bonds in the GGBS structure but also accelerates the depolymerization of its amorphous phase. On this basis, GGBS, as an active aluminosilicate precursor, gradually dissolves and releases reactive species such as Ca^2+^, SiO_4_^4−^, and [Al(OH)_4_]^−^. These ions migrate within the pore solution and recombine to form cementitious products dominated by C–S–H gel, thereby constructing the initial skeletal framework. Meanwhile, the continuously increasing Ca^2+^ concentration in the system provides necessary conditions for sulfate reactions. PG, as the sulfate source, releases SO_4_^2−^, which reacts with Ca^2+^ and aluminate species in the solution to form ettringite (AFt) crystals. AFt grows in needle-like or bundle-like morphologies within the pore space, effectively filling pores and bridging solid particles, thereby significantly improving structural integrity. The above processes can be described by the following main reaction equations:

(6) $$2C_{3}S\left(s\right)+7H_{2}O\left(l\right)\to C-S-H(s)+3Ca(OH{)}_{2}(aq)$$

(7) $$GGBS(s)+OH^{-}(aq)+H_{2}O(l)\to Ca^{2+}(aq)+SiO_{4}^{4-}(aq)+[Al(OH{)}_{4}{]}^{-}\left(aq\right)$$

(8) $$Ca^{2+}\left(aq\right)+SiO_{4}^{4-}\left(aq\right)+H_{2}O\left(l\right)\to C-S-H\;(s)$$

(9) $$3Ca^{2+}(aq)+2[Al(OH{)}_{4}{]}^{-}(aq)+3SO_{4}^{2-}(aq)+32H_{2}O(l)\to AFt(s)$$

During the above reaction process, the continuous formation of C–S–H gel provides the primary binding effect, while the growth of ettringite (AFt) crystals further strengthens the structure through pore filling and particle bridging. The synergistic interaction between these two phases significantly reduces porosity and refines the pore size distribution, leading to a denser and more stable microstructure. This dual mechanism of gel bonding and crystal filling jointly governs the development of the mechanical properties of the stabilized soil.

### 3.9. Limitations and Future Perspectives

This study systematically investigated the effects of the water-to-binder ratio on the workability, mechanical properties, and microstructural evolution of SGPC-based flowable solidified soil. A combination of FTIR, XRD, TG–DTG, SEM–EDS, and BET techniques was employed to preliminarily examine the formation of hydration products and changes in pore structure. The results indicate that appropriate control of the water-to-binder ratio can achieve a good balance between flowability and mechanical performance, while promoting matrix densification and strength development. However, this study still has certain limitations. First, it mainly focuses on the early-age performance evolution of the material, and a systematic evaluation of long-term durability has not yet been conducted. In particular, the service performance and degradation mechanisms under complex environmental conditions such as dry–wet cycles, freeze–thaw cycles, sulfate attack, and permeability remain unverified. Second, considering that the system incorporates industrial solid wastes such as alkaline slag and phosphogypsum, its environmental safety in practical engineering applications still requires further assessment, especially regarding the migration behavior of heavy metals or potentially harmful components under long-term leaching conditions. In addition, the economic and environmental benefit analysis is based on typical carbon emission factors and cost data, and a complete life cycle assessment (LCA) framework has not yet been established; therefore, the results are still subject to boundary limitations. Moreover, the setting behavior was characterized using the Vicat method, which is only suitable for comparative analysis under different water-to-binder ratios, while in practical engineering applications it is also influenced by particle gradation, skeleton formation, and construction disturbance. Meanwhile, the microstructural mechanism analysis is still mainly qualitative, and quantitative characterization of gel structure evolution and pore distribution remains insufficient. Furthermore, the present study is primarily based on laboratory conditions, lacking field validation and long-term in situ monitoring data. Future research should integrate in situ testing and practical engineering applications to further investigate the reaction mechanisms, long-term stability, and engineering applicability of multi-solid-waste synergistic systems.

## 4. Conclusions

Based on the systematic investigation into the influence of water-to-solid ratio (W/S) on the workability, mechanical properties, microstructural evolution, and environmental–economic performance of SGPC-stabilized flowable soil, the main findings of this study can be summarized as follows:

(1) Water-to-solid ratio significantly affected both workability and strength performance of SGPC-stabilized flowable soil. The 28-day UCS decreased monotonically from 1585.55 kPa (SGPC41) to 860.15 kPa (SGPC49) as W/S increased. Although SGPC41 exhibited the highest strength, its initial flowability (152 mm) did not satisfy practical requirements. SGPC43 achieved a UCS of 1450.13 kPa together with an initial flowability of 173 mm. Under the investigated experimental conditions, W/S = 0.43 demonstrated a favorable balance between strength performance and workability among the tested mixtures.

(2) Increasing W/S reduced hydration product formation and promoted pore development. TG–DTG results showed that the total mass loss at 800 °C decreased from 20.86% in SGPC41 to 7.76% in SGPC49 with increasing W/S, indicating a reduction in hydration-product content. BET analysis indicated that cumulative pore volume increased from approximately 0.10 cm^3^·g^−1^ to 0.16 cm^3^·g^−1^. SEM observations further revealed a less continuous gel matrix and enhanced pore connectivity at high W/S.

(3) SGPC exhibited considerable environmental and economic advantages while maintaining competitive strength performance. At W/S = 0.43, SGPC43 achieved 87.45% of the 28-day UCS of cement-treated soil (1450.13 vs. 1658.26 kPa). Meanwhile, embodied carbon emissions decreased by 74.7% (235 vs. 930 kg CO_2_-e/t), and material cost was reduced from 110 to 53 USD/t, demonstrating substantial sustainability benefits.

(4) pH measurements showed that all specimens maintained highly alkaline conditions (11.4–12.2) during curing. XRD and TG–DTG analyses confirmed the formation of AFt and C–(A)–S–H phases. The results suggest that hydration development in SGPC was governed by the synergistic interaction among SR, PG, and cement, which contributed to hydration-product generation and microstructure densification.

## Figures and Tables

**Table 1:** Basic physical properties of the WS.

Physical Indicator	Water Content (%)	Liquid Limit (%)	Plastic Limit (%)	Plasticity Index	Specific Gravity (–)	Maximum Dry Density (g/cm^3^)	pH
Value	46.52	41.39	21.61	19.78	2.72	1.83	6.68

Note: Specific gravity is a dimensionless parameter.

**Table 2:** Experimental design and mixture proportions.

Mix ID	SR Content (% of WS)	GGBS Content (% of WS)	PG Content (% of WS)	PC Content (% of WS)	W/S Ratio	Curing Age (d)
SGPC41	1.12	9.072	1.008	2.8	0.41	3, 7, 14, 28
SGPC43	1.12	9.072	1.008	2.8	0.43	
SGPC45	1.12	9.072	1.008	2.8	0.45	
SGPC47	1.12	9.072	1.008	2.8	0.47	
SGPC49	1.12	9.072	1.008	2.8	0.49	
C43	1.12	9.072	0	14	0.43	

Note: In Table 2, C43 is the designated pure cement control group; SGPC content and PC content are expressed as percentages of the dry mass of WS.

**Table 3:** Main nomenclature and abbreviations adopted in this study.

Symbol	Meaning	Symbol	Meaning	Symbol	Meaning
C_3_S	Tricalcium silicate	Ca(OH)_2_	Calcium hydroxide	GGBS	Ground granulated blast furnace slag
C_2_S	Dicalcium silicate	SiO_2_	Silicon dioxide	PG	Phosphogypsum
C_3_A	Tricalcium aluminate	Al_2_O_3_	Aluminum oxide	PC	Ordinary Portland cement
C–S–H	Calcium silicate hydrate gel	SO_3_	Sulfur trioxide	SR	Soda residue
CaO	Calcium oxide	AFt	Ettringite	WS	Waste soil
SEM	Scanning electron microscopy	EDS	Energy dispersive spectroscopy	SGPC	Multi-solid-waste composite cementitious system
XRD	X-ray diffraction	TG/DTG	Thermogravimetric analysis	BET	N_2_ adsorption–desorption specific surface area analysis

**Table 4:** Embodied carbon factors and material prices of raw materials.

Material	Embodied Carbon Factor (kg CO_2_-e/kg)	Unit Price (USD/t)	Data Source
PC	0.93	110	Hammond et al. [91]
GGBS	0.07	45	Bheel et al. [92]
PG	0.02 (0.01–0.03)	15	LCA-based conservative estimate [93,94,95]
SR	0.03 (0.01–0.05)	10	LCA-based conservative estimate [93,94,95]

**Table 5:** Environmental and economic performance of binder systems.

Binder System	PC (%)	GGBS (%)	PG (%)	SR (%)	Embodied Carbon (kg CO_2_-e/t)	Material Cost (USD/t)
C43	100	0	0	0	930	110
SGPC43	20	64.8	7.2	8	235	53

## Data Availability

The original contributions presented in this study are included in the article. Further inquiries can be directed to the corresponding author.
